# Polymethoxyflavone Apigenin-Trimethylether Suppresses LPS-Induced Inflammatory Response in Nontransformed Porcine Intestinal Cell Line IPEC-J2

**DOI:** 10.1155/2015/673847

**Published:** 2015-06-09

**Authors:** Orsolya Farkas, Orsolya Palócz, Erzsébet Pászti-Gere, Péter Gálfi

**Affiliations:** Department of Pharmacology and Toxicology, Faculty of Veterinary Science, Szent István University, István utca 2, Budapest 1078, Hungary

## Abstract

The *in vitro* anti-inflammatory effect of apigenin and its trimethylated analogue (apigenin-trimethylether) has been investigated in order to evaluate whether these flavonoids could attenuate LPS-induced inflammation in IPEC-J2 non-transformed intestinal epithelial cells. Levels of IL-6, IL-8, TNF-*α*, and COX-2 mRNA were measured as a marker of inflammatory response. The extracellular H_2_O_2_ level in IPEC-J2 cells was also monitored by Amplex Red assay. Our data revealed that both compounds had significant lowering effect on the inflammatory response. Apigenin (at 25 *μ*M) significantly decreased gene expression of IL-6 in LPS-treated cells, while apigenin-trimethylether in the same concentration did not influence IL-6 mRNA level. Both apigenin and apigenin-trimethylether reduced IL-8 gene expression significantly. TNF-*α* mRNA level was decreased by apigenin-trimethylether, which was not influenced by apigenin. Treatment with both flavonoids caused significant reduction in the mRNA level of COX-2, but the anti-inflammatory effect of the methylated analogue was more effective than the unmethylated one. Furthermore, both flavonoids reduced significantly the level of extracellular H_2_O_2_ compared to the control cells. In conclusion, the methylated apigenin analogue could avoid LPS-induced intestinal inflammation and it could be applied in the future as an effective anti-inflammatory compound.

## 1. Introduction

Flavonoids are naturally occurring polyphenolic compounds which are part of the regular human diet, because of their presence in vegetables, fruits, and beverages such as wine, coffee, and tea. A broad spectrum of beneficial effects (e.g., anticancer, antibacterial, and anti-inflammatory) is attributed to these molecules [1–3]. Many of the positive biological actions of flavonoids have been assigned to their antioxidant properties. However, there is an emerging view that flavonoids and their* in vivo* metabolites do not act only as conventional hydrogen-donating antioxidants but could modulate protein kinase signalling pathways in cells, influencing transcription factor nuclear factor kappa B (NF-*κ*B) [2].

Polymethoxylated flavones (PMFs) or polymethoxyflavones are flavones substituted with two or more methoxy groups. They are coming into center of interest more and more due to their documented wide spectrum of biological activity including anti-inflammatory, anticarcinogenic, and antiatherogenic properties [4–7]. Some of the polymethoxylated citrus flavonoids have also demonstrated antiproliferative properties in preliminary studies [8, 9]. A novel tangeretin derivative, 5-acetyl-6,7,8,4′-tetramethylnortangeretin, inhibited MCF-7 breast cancer cell growth through both caspase-dependent intrinsic apoptosis and caspase-independent apoptosis pathways [10].

The antiproliferative effects of methoxylated versus hydroxylated flavones were directly compared in SCC-9 human oral squamous carcinoma cells [6]. Apigenin-trimethylether was about eight times more potent than apigenin, one of the most studied hydroxyflavones. More recent research data demonstrated that citrus PMFs are directly associated with the inhibition of enzymes involved in the inflammation [11–13]. From previous reports, it seemed that flavonoids with free hydroxyl groups are more physiologically active than their methylated derivatives because of their stronger free radical scavenging activity. Hence, hydroxylated polyphenols scavenge free radical species better, whereas fully methoxylated flavonoids can also effectively inhibit the enzymes like inducible nitric oxide synthase (iNOS) and NADPH oxidase that generate free radicals like NO and superoxide anion [11, 14]. Nobiletin, the most studied element of the PMF family, has been shown to inhibit the production of prostaglandin E2 (PGE2) in human synovial fibroblasts by selectively downregulating COX-2 [13, 15]. Gene expression of proinflammatory cytokines, such as IL-1*α*, IL-1*β*, TNF-*α*, and IL-6, was found downregulated by nobiletin, tangeretin, and 3,5,6,7,8,3′,4′-heptamethoxyflavone [15, 16]. It has been shown in an LPS-induced mouse macrophage model that nobiletin, tangeretin, and their derivatives moderately attenuated iNOS and COX-2 gene expression [16, 17]. Nevertheless, the available information about the biological effect of methoxylated flavonoids is not fully explored.

Methoxyflavones originate from plant-derived foods, especially from citrus peel, and could be released from hydroxyflavones by the catechol-O-methyltransferase [18] in the enterocytes. Due to their increased bioavailability [6], methoxylated flavones are more able to induce beneficial effects* in vivo* as compared to their hydroxylated analogs.

Intestinal epithelial cells play an important role in the innate immune response against pathogenic bacteria. Besides acting as a physical barrier, previous studies suggest that epithelial cells also have significant role in generating signals by the production of several cytokines, chemokines, and other signalling molecules [19–21]. The phosphoglycolipid LPS, component of the outer membrane in Gram-negative bacteria, is recognized by epithelial toll-like receptor-4 (TLR-4) [22]. Ligation of TLR initiates a signalling cascade that results in the activation of the transcription factor NF-*κ*B and subsequent upregulation of costimulatory molecules as well as inflammatory cytokines and chemokines [23]. NF-*κ*B also regulates the expression of COX-2, affecting the production of prostaglandins.

*In vitro* gut models offer a suitable alternative to* in vivo* animal experiments. Cancerous cell lines such as Caco-2 and HT-29 are widely used for this tool. However, the major disadvantage of cell lines originated from cancer tissues is that their glycosylation pattern, proliferation rate, and colonisation ability significantly differ from healthy tissues. The nontransformed porcine intestinal epithelial cell line IPEC-J2, originally isolated from jejunal epithelia of a neonatal unsuckled piglet, models* in vivo *structure and function of the small intestine more closely than colon tumorigenic cell lines. This cell line forms polarized monolayers with high transepithelial electrical resistance when cultured on 0.4 *μ*m pore-size filters, developing apical and basolateral part [24]. Because of the abovementioned facts, IPEC-J2 cell line is a realistic and representative tool for mimicking the human as well as the pig small intestine. It can be good tool for pharmacology research, toxicity, microbiology, bioavailability, and metabolism studies in the field of human as well as veterinary medicine and food science [25].

**Figure 1:**
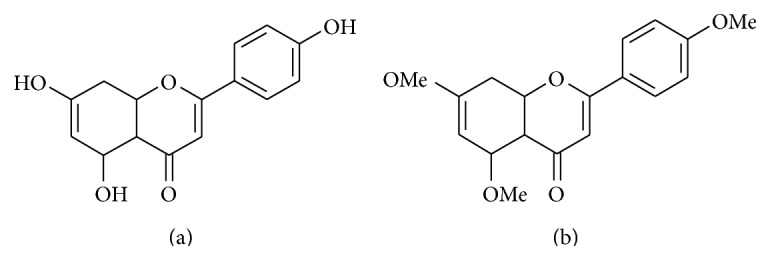
Structure of apigenin (3′,4′,5-trihydroxyflavone) (a) and apigenin-trimethylether (3′,4′,5-trimethoxyflavone) (b).

The main aim of this study is to investigate the possible protective effects of methoxyflavones in intestinal epithelial cells under the condition of inflammation. Apigenin (3′,4′,5-trihydroxyflavone, Figure 1(a)), a well-studied and abundant flavonoid, and their methylated analogue (3′,4′,5-trimethoxyflavone, Figure 1(b)) were chosen as test compounds in order to study the abovementioned subject. This is the first report, which describes the effect of apigenin in intestinal inflammation using nontransformed intestinal epithelial cell line. Moreover, comparison of the anti-inflammatory effect of an unmethylated flavonoid and its methylated analogue in a nontransformed intestinal cell model was also performed at first time.

## 2. Materials and Methods

### 2.1. Chemicals

Apigenin (≥97%), dimethyl sulfoxide (≥99.7%, sterile-filtered, BioReagent), LPS (derived from* Salmonella enterica *ser. Typhimurium), and H_2_O_2_ (30%) were purchased from Sigma-Aldrich (Steinheim, Germany). Apigenin-trimethylether (≥99%) was from INDOFINE Chemical Company (Hillsborough, NJ, USA).

### 2.2. Cell Line and Culture Conditions

The nontransformed porcine intestinal epithelial cell line IPEC-J2, originally isolated from jejunal epithelia of a neonatal unsuckled piglet [24], was a kind gift from Dr. Jody Gookin, Department of Clinical Sciences, College of Veterinary Medicine, North Carolina State University, Raleigh, NC, USA. IPEC-J2 cells were grown and maintained in complete medium, which consisted of a 1 : 1 mixture of Dulbecco's Modified Eagle's Medium and Ham's F-12 Nutrient Mixture (DMEM/F12) (plain medium) supplemented with 5% foetal bovine serum (FBS), 5 *μ*g/mL insulin, 5 *μ*g/mL transferrin, 5 ng/mL selenium, 5 ng/mL epidermal growth factor, and 1% penicillin-streptomycin (all from Fisher Scientific, Loughborough, UK). Cells were grown at 37°C in a humidified atmosphere of 5% CO_2_. Cell cultures were tested by PCR and they were found to be free of* Mycoplasma *contamination.

For the experiments, IPEC-J2 cells between passages 42 and 48 were seeded onto six-well Transwell polyester membrane inserts (Corning Inc., Corning, NY, USA), the latter coated with 8 *μ*g/cm^2^ rat tail collagen type I (Sigma-Aldrich, Steinheim, Germany), at a density of 1.5 × 10^5^ cells/mL (the volume of complete medium was 1.5 mL on the apical side and 2.5 mL on the basolateral side per well according to the manufacturer's instructions). Cells were allowed to adhere for 24 h before being washed and refed every other day until confluence was reached. Transepithelial electrical resistance (TEER) measurement of monolayers was performed on alternate days after seeding, from days 5 to 21 of culture, using an EVOM Epithelial Tissue Volt/Ohmmeter (World Precision Instruments, Berlin, Germany).

### 2.3. Neutral Red Uptake Assay for Cell Viability

Influence of apigenin and apigenin-trimethylether on the viability of enterocytes in different concentrations (25, 50, and 100 *μ*M) was tested. Flavonoids were dissolved in DMSO and diluted in cell culture medium. The final concentration of DMSO at the cells was 0.1%. In control experiments, this concentration did not show any effects on the measured parameters. IPEC-J2 cells were seeded in a 96-well plate and incubated with polyphenols for 1, 2, 4, and 24 h, respectively. Viability of IPEC-J2 cells was measured 24 hours after treatment by Neutral Red uptake assay as described by Repetto et al. [26].

### 2.4. Treatment of Cells with LPS

Before treatment, confluent monolayers of the IPEC-J2 cells were washed with plain medium. LPS solutions were prepared freshly prior to each experiment. LPS was added in plain medium at 10 *μ*g/mL on the apical side of the IPEC-J2 layer. After 1 h incubation with LPS and flavones, cells were washed with plain medium and cultured for additional 1 h for PCR studies. TEER measurements were performed both before and after the LPS treatment.

### 2.5. Measurement of Extracellular H_2_O_2_

Fluorescent ROS measurement was based on the detection of H_2_O_2_ using the Amplex Red Hydrogen Peroxide Assay Kit (Invitrogen, Molecular Probes) keeping the IPEC-J2 cells on the 96-well plate. In the presence of horseradish peroxidase (HRP), Amplex Red reacts with H_2_O_2_ in a 1 : 1 stoichiometry producing a highly fluorescent resorufin [27].

IPEC-J2 cells were treated with LPS in phenol-red free DMEM and the H_2_O_2_ concentrations in the medium were determined using the working solution of 100 *μ*M Amplex Red reagent and 0.2 U/mL HRP. H_2_O_2_ determination was also performed after 1 h LPS treatment immediately and 24 h incubation with phenol-red free DMEM. After 30 min incubation with the dye at room temperature the quantitative analyses of H_2_O_2_ contents were accomplished, the excitation wavelength was set at 560 nm, and emission was measured at 590 nm (Victor X2 2030 fluorometer, Perkin Elmer, Waltham, MA, USA).

### 2.6. Quantitative Real-Time PCR

One hour after the 1 h LPS treatment, culture medium was removed and 1 mL of ice-cold TRIzol reagent (Invitrogen, Carlsbad, CA, USA) was added to the IPEC-J2 samples. Samples were collected and kept at −80°C until further processing. Total RNA was isolated from the cells according to the manufacturer's instructions. To prevent DNA contamination, the isolated RNA (2 *μ*g) was treated with AMP-D1 DNase I (Sigma). Quantity *A*_260_/*A*_280_ and *A*_260_/*A*_230_ ratios of the extracted RNA were determined using a NanoDrop ND-1000 Spectrophotometer (Thermo Scientific, Wilmington, USA). Quality and quantity control of the isolated RNA were carried out both before and after the DNase treatment.

Synthesis of the first strand of cDNA from 1000 ng of total RNA was achieved using RevertAid H Minus First Strand cDNA Synthesis Kit (Fermentas, St. Leon-Roth, Germany) according to the manufacturer's recommendations, using the random hexamer as a priming method. Quantitative real-time PCR (qRT-PCR) was performed using the iQ SYBR Green Supermix kit (BioRad, Hercules, CA, USA) on the MiniOpticon System (BioRad). The cDNA was diluted 5-fold, before equal amounts were added to duplicate qRT-PCR reactions. Tested genes of interest were IL-6, IL-8, TNF-*α*, COX-2, and Hsp70. Hypoxanthine phosphoribosyl transferase (HPRT) and Cyclophilin-A (CycA) were used as reference genes. Primer sequences are listed in Table 1. For each PCR reaction, 2.5 *μ*L cDNA was added directly to a PCR reaction mixture and set to a final volume of 25 *μ*L, containing 1x concentrated iQ SYBR Green Supermix and 0.2 *μ*M of the appropriate primers. The thermal profile for all reactions was 3 min at 95°C, then 40 cycles of 20 sec at 95°C, 30 sec at 60°C, and 30 sec at 72°C. At the end of each cycle, the fluorescence monitoring was set for 10 seconds. Each reaction was completed with a melting curve analysis to ensure the specificity of the reaction. In order to determine the efficiencies of the PCR reactions, standard curves were obtained for each target and reference gene, using serial dilutions of a reference cDNA. Real-time PCR efficiencies (*E*) were calculated according to the equation: *E* = 10(−1/slope). To determine the stability of the reference genes, the geNorm (version 3.5) was used.

### 2.7. Statistics

Relative gene expression levels of the genes of interest were calculated by the Relative Expression Software Tool (REST) 2009 Software (Qiagen GmbH, Hilden, Germany). Statistical analysis of other data was performed with STATISTICA 10 software (StatSoft Inc., Tulsa, USA). Differences between means were evaluated by one-way ANOVA, with data of normal distribution, and homogeneity of variances was confirmed. To compare treated groups to controls we used Dunnett post-hoc test. For the comparison of different treatments we used Fisher LSD test. Level of significance was set at *P* < 0.05. All values were expressed as means ± standard deviations.

## 3. Results

### 3.1. Viability of IPEC-J2 Cells after Flavonoid Treatment

**Figure 2:**
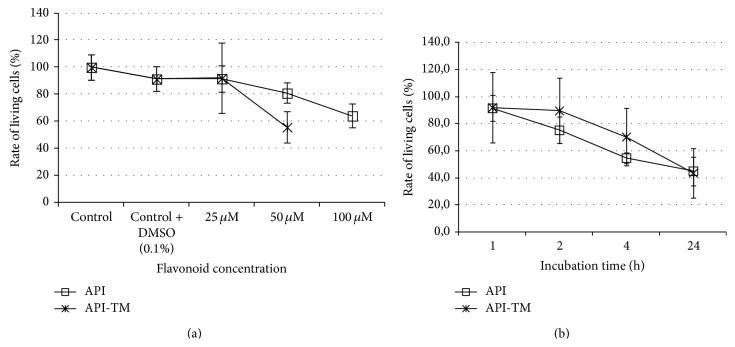
Rate of living IPEC-J2 cells after apigenin and apigenin-trimethylether treatment. Cell viability was tested using Neutral Red uptake method. Effect of flavones on cell viability was studied in a dose- (a) and time-dependent (b) manner. Data are shown as means + standard deviations.

Viability of IPEC-J2 cells was monitored after apigenin and apigenin-trimethyether treatment, respectively (see Figures 2(a) and 2(b)). Neutral Red uptake assay showed that there was no significant difference in number of viable IPEC-J2 cells incubated with plain DMEM containing 0.1% DMSO. At 25 *μ*M treatment dose in case of both flavones for 1 h did not reduce the number of viable enterocytes significantly. More than 44% of the IPEC-J2 cells were killed by 1 h treatment when apigenin-trimethylether was applied at 50 *μ*M concentrations, while incubation with 100 *μ*M destroyed 55.9 ±8.53% of cells. After 1 h exposure of 50 *μ*M and 100 *μ*M apigenin, reduced viability of IPEC-J2 cells was detected (rate of living cells decreased to 80.9 ± 7.49% and 64.1 ± 8.53%, resp., compared to the control samples). On the basis of the abovementioned data, it seemed to be safe to use both flavones in 25 *μ*M concentration for further experiments. Viability was tested also on a time-dependent manner, using 25 *μ*M concentrations of polyphenols. Living cell rate decreased to 75.3 ± 9.9% after 2 h apigenin-trimethylether exposure, while 4 h and 24 h treatment greatly reduced number of living IPEC-J2 cells (rate of viable cells was 70.3 ± 20.1 and 43.6 ± 18.3, resp.). Reduced viability was observed when enterocytes were treated with 25 *μ*M apigenin for 4 h and 24 h, respectively (in preliminary studies rate of viable cells was 54.7 ± 3.84 and 45.0 ± 10.5, resp.). Therefore, 1 h treatment period was used in further experiments.

### 3.2. Inflammatory Response in IPEC-J2 Cells after LPS Treatment

In order to verify the integrity of the IPEC-J2 cell layer, TEER values between apical and basolateral compartment were measured. Experiments were performed with confluent polarized IPEC-J2 cells with high TEER values. The integrity of the cell monolayer was not altered after LPS treatment (data not shown).

Relative gene expressions of IL-6, IL-8, TNF-*α*, COX-2, and Hsp70 in LPS-triggered IPEC-J2 cells were determined. LPS treatment significantly increased TNF-*α* (*P* = 0.018), IL-6 (*P* = 0.044), and IL-8 (*P* = 0.001) relative gene expression levels. Significant upregulation of COX-2 gene was also observed after 1 h 10 *μ*g/mL LPS administration (*P* = 0.012). There was no alteration in the mRNA level of heat shock protein 70 (*P* = 0.375) after LPS treatment.

### 3.3. Effect of Apigenin and Apigenin-Trimethylether on the Relative Gene Expression of Cytokines TNF-*α*, IL-6, and IL-8 in LPS-Treated Enterocytes

**Figure 3:**
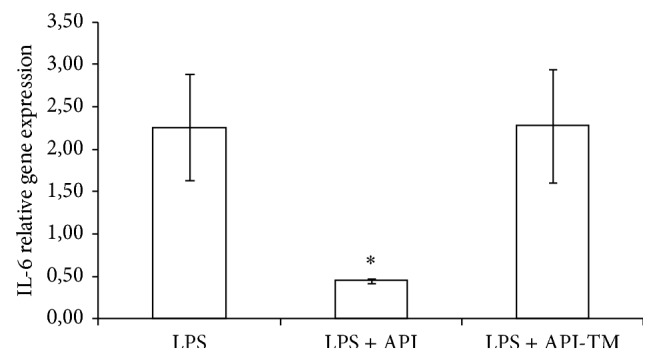
Relative gene expression of IL-6 in IPEC-J2 cells exposed to LPS treatment (at 1 *μ*g/mL; 1 h treatment). Effect of apigenin (25 *μ*M) and apigenin-trimethylether (25 *μ*M) on the IL-6 mRNA levels (*n* = 3-4/group; ^*^ *P* < 0.05). Data are shown as means + standard deviations. API = apigenin; API-TM = apigenin-trimethylether.

**Figure 4:**
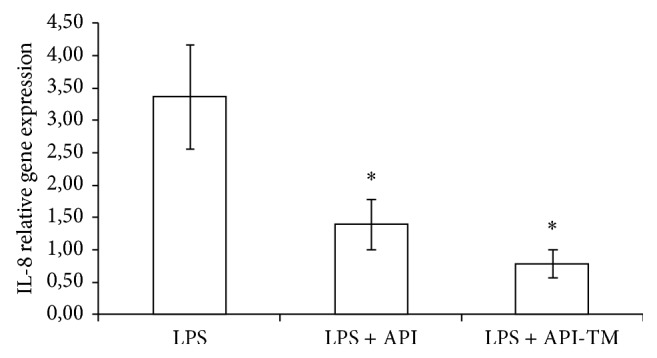
Relative gene expression of IL-8 in IPEC-J2 cells exposed to LPS treatment (at 1 *μ*g/mL; 1 h treatment). Effect of apigenin (25 *μ*M) and apigenin-trimethylether (25 *μ*M) on the IL-8 mRNA levels (*n* = 3-4/group; ^*^ *P* < 0.05). Data are shown as means + standard deviations. API = apigenin; API-TM = apigenin-trimethylether.

**Figure 5:**
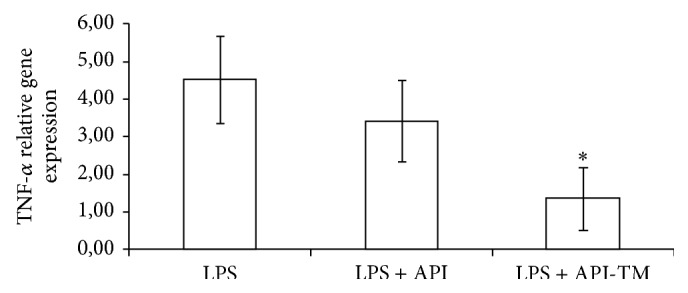
Relative gene expression of TNF-*α* in IPEC-J2 cells exposed to LPS treatment (at 1 *μ*g/mL; 1 h treatment). Effect of apigenin (25 *μ*M) and apigenin-trimethylether (25 *μ*M) on the TNF-*α* mRNA levels (*n* = 3-4/group; ^*^ *P* < 0.05). Data are shown as means + standard deviations. API = apigenin; API-TM = apigenin-trimethylether.

At 25 *μ*M apigenin treatment dose, relative gene expression of IL-6 significantly decreased compared to LPS-treated cells (*P* = 0.0348). Apigenin-trimethylether in the same concentration did not influence the IL-6 mRNA level. Both apigenin (*P* = 0.0009) and apigenin-trimethylether (*P* = 0.0014) caused significant reduction in the IL-8 gene expression. There was no significant difference in the IL-8 gene expression reducing effect of apigenin and its unmethylated analogue. TNF-*α* mRNA level was decreased by 25 *μ*M apigenin-trimethylether treatment (*P* = 0.0081), while apigenin did not suppress TNF-*α* mRNA level compared to the LPS-treated enterocytes. Figures 3–5 show the effect of flavonoids on proinflammatory cytokine gene expressions.

### 3.4. Cox-2 Relative Gene Expression

**Figure 6:**
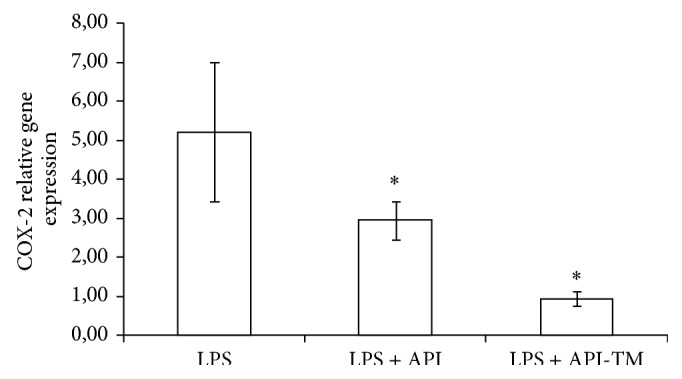
Relative gene expression of COX-2 in IPEC-J2 cells exposed to LPS treatment (at 1 *μ*g/mL; 1 h treatment). Effect of apigenin (25 *μ*M) and apigenin-trimethylether (25 *μ*M) on the COX-2 mRNA levels (*n* = 3-4/group; ^*^ *P* < 0.05). Data are shown as means + standard deviations. API = apigenin; API-TM = apigenin-trimethylether.

Apigenin and apigenin-trimethylether treatment caused significant reduction in the mRNA level of COX-2 (*P* = 0.0287, *P* = 0.0006) Effect of apigenin and apigenin-trimethylether was compared using one-way ANOVA (Fisher LSD test). It was shown that there is a significant difference between the effect of hydroxy and methoxy-analogue (*P* = 0.0264). The influence of apigenin and its trimethylated derivative on COX-2 relative gene expression could be seen in Figure 6.

### 3.5. Extracellular H_2_O_2_ Production in IPEC-J2 Cells after LPS and Flavonoid Treatment

**Figure 7:**
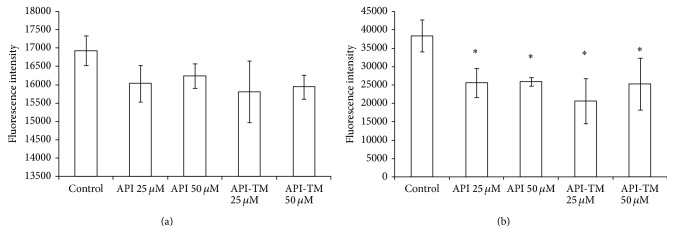
Level of extracellular H_2_O_2_ in IPEC-J2 cells exposed to flavonoid treatment (25 and 50 *μ*M; 1 h). Effect of apigenin and apigenin-trimethylether on the relative extracellular H_2_O_2_ levels (*n* = 3-4/group; ^*^ *P* < 0.05). Fluorescence measurement was performed by Amplex Red method. Fluorescence was detected immediately (a) and 24 h after (b) flavonoid treatment. Data are shown as means + standard deviations. API = apigenin; API-TM = apigenin-trimethylether.

Figure 7 shows the relative extracellular H_2_O_2_ level in IPEC-J2 cells after flavonoid treatment. LPS only at higher concentration (at 50 *μ*g/mL) increased the level of extracellular H_2_O_2_; however, IPEC-J2 cells are irreversibly damaged using LPS in this concentration (data not shown). Flavonoids showed different effects on the rate of H_2_O_2_ production, depending on the duration of incubation in DMEM after flavonoid treatment. In case of short time effect measurements (1 h incubation with flavonoids and detection immediately after the incubation period), neither apigenin nor its trimethylated analogue decreased mitochondrial H_2_O_2_ production rate effectively. Long time effects of flavonoid treatment (Amplex Red measurement was performed 24 h after the 1 h flavonoid incubation) were also studied. Extracellular H_2_O_2_ level was significantly decreased in case of both lower (25 *μ*M) and higher (50 *μ*M) concentration apigenin treatments. The same reducing effect was found, when 25 *μ*M and 50 *μ*M apigenin-trimethylether were applied.

## 4. Discussion

LPS, a major integral component of the outer membrane of Gram-negative bacteria, is widely used to induce inflammation in case of several cell types, including small intestinal cell lines such as IPEC-J2. NF-*κ*B activation resulted in the expression of a wide range of genes, many of which are markedly upregulated in response to microbial infection and inflammation. Some of these are cytokines and chemokines (IL-1, IL-6, IL-8, and TNF-*α*) and inflammatory enzymes such as COX-2. The inhibition of signal transduction pathways at any point of the inflammatory cascade reduces the production of these proteins, which modulates the inflammation locally [33].

Considering the literature [21, 34, 35] and our previous results, detection of relative gene expression of proinflammatory cytokines in IPEC-J2 intestinal epithelial cells was optimal after 1 and 2 hours [36, 37]. In our experiments, 1 h incubation of IPEC-J2 cells with LPS (and further 1 h incubation with medium) resulted in significantly high relative IL-6, IL-8, and TNF-*α* as well as COX-2 gene expression level. Relative expression of the abovementioned genes was tested 4 h after LPS treatment as well, and those results did not differ significantly from the expression data from 1 h LPS treatment. Expression of TLR-4, a key element of the activation of NF-*κ*B, in IPEC-J2 cells after LPS treatment was also measured in our previous study [37]. No difference in the TLR-4 expression was detected compared to the control samples, using different concentrations, incubation times, and different types of LPS. Our data agrees with the results of other studies [23, 38, 39], that epithelial cell lines often show a low level expression of TLR-4, explained by the fact that intestinal epithelial cells are relative resistant to the permanent exposure to Gram-negative commensal bacteria.

The* in vitro* anti-inflammatory effect of apigenin was studied in many cases. However, these reports were performed using cancerous cell lines or immune cells. LPS-stimulated human peripheral blood mononuclear cells were cultured in the presence of apigenin, and TNF-*α*, IL-1*β*, and IL-6 were measured in the cell culture supernatant [40]. Apigenin inhibited proinflammatory cytokine production dose-dependently. Murine macrophage cells and mice were treated with LPS and herbal constituents by Smolinski and Pestka [41]. IL-6 and TNF-*α* were measured from serum and supernatant by ELISA. All three constituents including apigenin inhibited LPS-induced IL-6 and TNF-*α* production in murine macrophage culture. Inhibition of these two cytokines in mice did not display the same patterns of inhibition as cell culture data.* In vitro* cotreatment with apigenin reduced LPS-induced IL-6, but not TNF-*α* production. Cotreatment with 3.7 and 37 mM of apigenin significantly reduced IL-6, while inhibition of TNF-*α* was not observed. However, a study of Mastuda et al. [42] showed that apigenin, at IC50 = 5.3 mM, inhibited antigen-IgE-mediated TNF-*α* secretion in RBL-2H3 mast cells. Apigenin also inhibited TNF-*α* production as well as iNOS expression and NO production in LPS-activated macrophages, and this effect has been associated with the inhibition of the NF-*κ*B pathway [43]. Apigenin inhibited TNF-*α* secretion in a concentration-dependent manner. It shows only a slight effect on TNF-*α* release that did not reach 25 *μ*M concentration, whereas apigenin decreases TNF-*α* release by 41.6% at 50 *μ*M. In our model, apigenin (25 *μ*M) treatment reduced both IL-6 and IL-8 mRNA level significantly compared to the LPS-treated group, while it did not affect TNF-*α*. A potential reason for the differences in results could be the differences in stimuli and cell types used.

In a study of Wang and Huang [44] the anti-inflammatory effects of apigenin in* Helicobacter pylori*-infected human stomach adenocarcinoma cells (MKN45) were investigated and expression of I-*κ*B-a, COX-2, and proinflammatory cytokines was measured. Apigenin treatment (9.3–74 *μ*M) significantly increased I-*κ*B-a expression and thus inhibited NF-*κ*B activation. Expression of IL-8, IL-6, and COX-2 was significantly decreased. Apigenin inhibited the production of NO and PGE2 by suppressing the expression of iNOS and COX-2 protein in murine microglia cell line model [45]. Moreover, it suppressed p38 mitogen-activated protein kinase and c-Jun N-terminal kinase (JNK) phosphorylation without affecting the activity of extracellular signal-regulated kinase (ERK).

In terms of anti-inflammatory effect, only a few studies have reported methoxyflavones. The most studied methoxylated flavones originated from citrus peel.* Citrus aurantium* L. extract containing nobiletin, naringin, and hesperidin inhibited the proinflammatory mediators including cytokines, COX-2, and iNOS by blocking NF-*κ*B and mitogen-activated protein kinase (MAPK) signalling in LPS-stimulated macrophages. The molecular mechanism was associated with inhibition of the phosphorylation of I-*κ*B-a and nuclear translocation of the NF-*κ*B p-65 as well as phosphorylation of MAPK by flavonoids [46]. Contrarily, a formulated product from citrus peel extract (Gold Lotion) inhibited the gene expression of iNOS, but not COX-2 in a mouse skin inflammatory model. These differential effects may be explained due to the degree of dependency of iNOS and COX-2 promoters on various transcription factors [47]. Nobiletin has been shown to significantly suppress the activation of activator protein 1, NF-*κ*B, and cAMP response element-binding protein (CREB) [13].

During and Larondelle [48] studied whether methoxylated flavones versus their unmethylated analogs can modulate the intestinal inflammatory response. After IL-1*β* stimulus of Caco-2 cells, the anti-inflammatory activity of apigenin, chrysin, luteolin, and quercetin was investigated. Their results indicate that methoxylation of chrysin improves its anti-inflammatory effect significantly, probably through a more explicit inhibition of the NF-*κ*B signaling pathway. Nevertheless, the abovementioned effect was not observed in case of other flavonoids. Furthermore, neither apigenin nor its methylated analogue reduced IL-8 level significantly, but IL-6 and PGE-2 concentration were reduced by both compounds. It seems that hydroxyflavones and their methoxylated analogues share the same signal transduction pathways; however, methoxyflavones needs further investigations.

Oxidative stress is defined as an imbalance between production of free radicals and reactive metabolites, referred to as reactive oxygen species (ROS), and their elimination by protective mechanisms, so-called antioxidants [49]. This loss of equilibrium leads to damage of important biomolecules, with potential impact on the whole organism. Oxidative stress could activate a variety of transcription factors including NF-*κ*B and lead to chronic inflammation. Flavonoid antioxidants could protect cells against the damaging effects of ROS [50].

In this study, some oxidative stress markers were also followed. It was found that neither the mRNA level of Hsp70 nor level of extracellular H_2_O_2_ was affected by 10 *μ*g/mL LPS treatment. LPS in higher concentration (50 *μ*g/mL) increased extracellular H_2_O_2_ level significantly; however, IPEC-J2 cells are irreversibly damaged (data not shown).

Nevertheless, effect of flavones on oxidative stress markers is worthy to be investigated, because they could act not only as antioxidants but also as prooxidants; that is, they are potential inducers of oxidative stress. The prooxidant potential of apigenin was studied by Miyoshi et al. [51]. Flow cytometric measurements and immunoblotting showed the intracellular accumulations of ROS and protein carbonyls in the cells treated with apigenin in a dose-dependent manner. In HL-60 cells treated with 50 *μ*M apigenin for 1 h, ROS level was 3.5-fold increased compared to control cells. Structure-activity relationship data suggested that presence of 4-hydroxy group and also the absence of 3-and 3′-hydroxy groups are important for prooxidant effect. Treatment cells with 25 *μ*M apigenin did not affect the extracellular ROS status. On the basis of our measurements in IPEC-2 intestinal epithelial cells, no prooxidant effect was observed. Moreover, extracellular H_2_O_2_ level in IPEC-J2 cells was significantly decreased 24 h after flavonoid treatment in case of both apigenin and apigenin-trimethylether. Deng et al. [52] compared the cytoprotective effect of methylated polyphenols and their unmethylated analogues. They found that methylation largely reduced chemical antioxidant capacity, but methylated flavons can still effectively protect lymphocytes from hydrogen peroxide-induced cytotoxicity.

The biological fate of the flavonoids is very complex, and it is dependent on a large number of parameters. Different activities could be influenced by the different absorption rate and metabolism. Absorption and metabolism studies of hydroxyflavones and methoxyflavones have been investigated primarily in cancer studies [6, 53]. Caco-2 cell model was established to test the absorption as well as permeability of polymethoxyflavones in the human intestine. Three compounds (3′-hydroxy-5,6,7,4′-tetramethoxyflavone, 3,5,6,7,8,3′,4′-heptamethoxyflavone, and 3-hydroxy-5,6,7,8,3′,4′-hexamethoxyflavone) showed very high permeability [54, 55]. The membrane transporters as well as metabolic enzymes could influence the cellular availability of polyphenols, affecting their anticancer potential. Nevertheless, absorptive and metabolism properties of these compounds could influence their anti-inflammation activities in the same way.

In conclusion, methylated flavonoids including apigenin-trimethylether may be useful tools in the treatment of intestinal inflammations in human as well in veterinary medicine. Future perspectives should include understanding the molecular basis for inhibitory effects of these compounds on proinflammatory cytokine gene expression and the role of altered absorption and metabolism.

## Figures and Tables

**Table 1:** Sequence of primer sets used for quantitative real-time.

Gene symbol	Accession number	Primer sequences	Product size (bp)	Efficiency	*R* ^2^	Reference
IL-8	NM_213867	F 5′-AGAGGTCTGCCTGGACCCCA-3′	126	1.972	0.999	[28]
		R 5′-GGGAGCCACGGAGAATGGGT-3′				

IL-6	NM_214399	F 5′-TTCACCTCTCCGGACAAAAC-3′	122	1.970	0.995	[29]
		R 5′-TCTGCCAGTACCTCCTTGCT-3′				

TNF-*α*	NM_214022	F 5′-TTCCAGCTGGCCCCTTGAGC-3′	146	1.873	0.982	[30]
		R 5′-GAGGGCATTGGCATACCCAC-3′				

COX-2	NM_214321	F 5′-AGAAGCGAGGACCAGCTTTC-3′	215	1.905	0.981	NCBI/Primer-Blast
		R 5′-AAAGCGGAGGTGTTCAGGAG-3′				

Hsp70	NM_001123127	F 5′-GCCCTGAATCCGCAGAATA-3′	152	2.0	0.993	[31]
		R 5′-TCCCCACGGTAGGAAACG-3′				

CycA	NM_214353	F 5′-GCGTCTCCTTCGAGCTGTT-3′	160	1.907	0.998	[30]
		R 5′-CCATTATGGCGTGTGAAGTC-3′				

HPRT	NM_001032376	F 5′-GGACTTGAATCATGTTTGTG-3′	91	1.963	0.997	[32]
		R 5′-CAGATGTTTCCAAACTCAAC-3′				

## Abbreviations

LPS: Lipopolysaccharide
IL-6: Interleukin-6
IL-8: Interleukin-8
TNF-*α*: Tumor necrosis factor alpha
COX-2: Cyclooxygenase 2.
